# Cloacal swabs and alcohol bird specimens are good proxies for compositional analyses of gut microbial communities of Great tits (*Parus major*)

**DOI:** 10.1186/s42523-020-00026-8

**Published:** 2020-03-17

**Authors:** Kasun H. Bodawatta, Katerina Puzejova, Katerina Sam, Michael Poulsen, Knud A. Jønsson

**Affiliations:** 1 Natural History Museum of Denmark, University of Copenhagen, Copenhagen, Denmark; 2 Biology Centre of Czech Academy of Sciences, Institute of Entomology, Branisovska 31, Ceske Budejovice, Czech Republic; 3 Faculty of Science, University of South Bohemia, Branisovska 1760, Ceske Budejovice, Czech Republic; 4 Section for Ecology and Evolution, Department of Biology, University of Copenhagen, Copenhagen, Denmark

**Keywords:** Non-invasive sampling, Museum alcohol bird collections, Microbiomes, Passeriformes, Digestive tract microbiota

## Abstract

**Background:**

Comprehensive studies of wild bird microbiomes are often limited by difficulties of sample acquisition. However, widely used non-invasive cloacal swab methods and under-explored museum specimens preserved in alcohol provide promising avenues to increase our understanding of wild bird microbiomes, provided that they accurately portray natural microbial community compositions. To investigate this assertion, we used 16S rRNA amplicon sequencing of Great tit (*Parus major*) gut microbiomes to compare 1) microbial communities obtained from dissected digestive tract regions and cloacal swabs, and 2) microbial communities obtained from freshly dissected gut regions and from samples preserved in alcohol for 2 weeks or 2 months, respectively.

**Results:**

We found no significant differences in alpha diversities in communities of different gut regions and cloacal swabs (except in OTU richness between the dissected cloacal region and the cloacal swabs), or between fresh and alcohol preserved samples. However, we did find significant differences in beta diversity and community composition of cloacal swab samples compared to different gut regions. Despite these community-level differences, swab samples qualitatively captured the majority of the bacterial diversity throughout the gut better than any single compartment. Bacterial community compositions of alcohol-preserved specimens did not differ significantly from freshly dissected samples, although some low-abundant taxa were lost in the alcohol preserved specimens.

**Conclusions:**

Our findings suggest that cloacal swabs, similar to non-invasive fecal sampling, qualitatively depict the gut microbiota composition without having to collect birds to extract the full digestive tract. The satisfactory depiction of gut microbial communities in alcohol preserved samples opens up for the possibility of using an enormous resource readily available through museum collections to characterize bird gut microbiomes. The use of extensive museum specimen collections of birds for microbial gut analyses would allow for investigations of temporal patterns of wild bird gut microbiomes, including the potential effects of climate change and anthropogenic impacts. Overall, the utilization of cloacal swabs and museum alcohol specimens can positively impact bird gut microbiome research to help increase our understanding of the role and evolution of wild bird hosts and gut microbial communities.

## Background

Appropriate sample collection and preservation methods are important when investigating symbioses between hosts and their gut microbes [1–4]. Although the use of entire digestive tracts of freshly obtained specimens is ideal to characterize and capture the full diversity and relative abundances of bacteria residing within guts [2, 5–7], the acquisition of full digestive tracts is often problematic in many vertebrate groups. This is especially true in wild birds and mammals due to permit limitations and ethical issues [8]. Consequently, many mammalian and avian microbiome studies utilize non-invasive methods (e.g., feces or cloacal swabs) [2, 9–11], which also provide opportunities to explore rare and endangered animals without negatively affecting host populations [12–15].

Most bird microbiome studies have utilized feces (e.g., [11, 13, 14, 16–20]) or cloacal swabs (e.g., [21–29]), but a handful of studies have demonstrated that bacterial communities are compartmentalized across different digestive tract regions [2, 5, 30, 31]. This questions whether non-invasive methods only capture fecal and cloacal bacterial communities. The small number of studies that have investigated the validity of non-invasive sampling methods to characterize bird gut microbiomes have shown that non-invasive methods consequently only represent gut microbiome compositions qualitatively [2, 32, 33]. This implies that we must be cautious in our conclusions about full gut community structure when utilizing non-invasive approaches, but at the same time emphasizes that non-invasive methods can identify symbiont lineages present within guts. Such validation studies have however only been conducted on a few non-passerine bird species [2, 32, 33], and we thus lack similar analyses from diverse clades such as the passerines. If easily-acquirable cloacal swabs adequately portray wild bird gut microbiomes, this could facilitate more studies and increase our understanding of wild bird-gut microbial symbioses [9, 34–36].

Another possible source of wild birds for digestive tract microbiome analyses is alcohol specimens stored in museums [37, 38]. European museums alone house more than 78,000 specimens [39], and around 45,000 specimens are housed in North America [40] and 22,000 in Australia and New Zealand [41]. Although sampling entire guts from alcohol specimens is not non-invasive, the utilization of these enormous unexplored collections would provide promising opportunities to investigate microbial communities, provided that they reliably portray gut microbial community compositions of freshly dissected digestive tracts.

To evaluate the accuracy of cloacal swabs and alcohol specimens to characterize gut microbiomes, we investigated gut microbial communities of Great tits (*Parus major*). First, we compared microbial communities (using 16S rRNA amplicon sequencing) from cloacal swabs with freshly dissected gut regions to validate the use of cloacal swabs to characterize the bacterial communities in the entire digestive tract. Secondly, we compared microbial communities in freshly dissected gut regions with gut sections that had been stored for two-weeks and two-months in alcohol to investigate the potential effect of alcohol preservation on gut microbial communities.

## Results

### Microbial community composition differences between freshly dissected gut regions and cloacal swabs

**Fig. 1:**
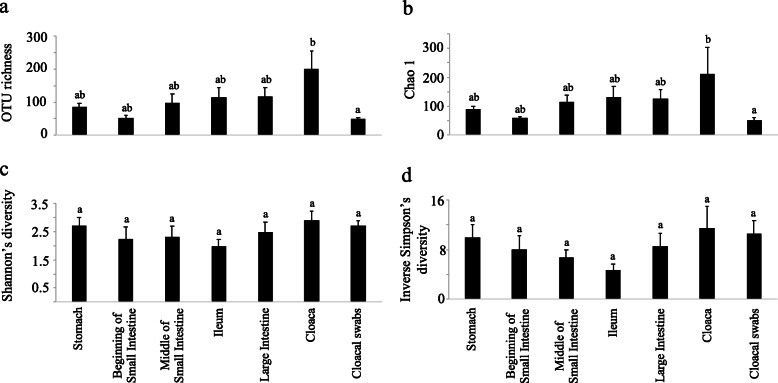
Average (**a**) OTU richness, (**b**) Chao 1 richness estimates, (**c**) Shannon’s diversity index and (**d**) Inverse Simpson’s diversity index of different digestive tract regions and cloacal swab microbial communities. Letters on top of each bar represent the results from the Tukey post hoc test and similar letters represent non-significant groups

**Fig. 2:**
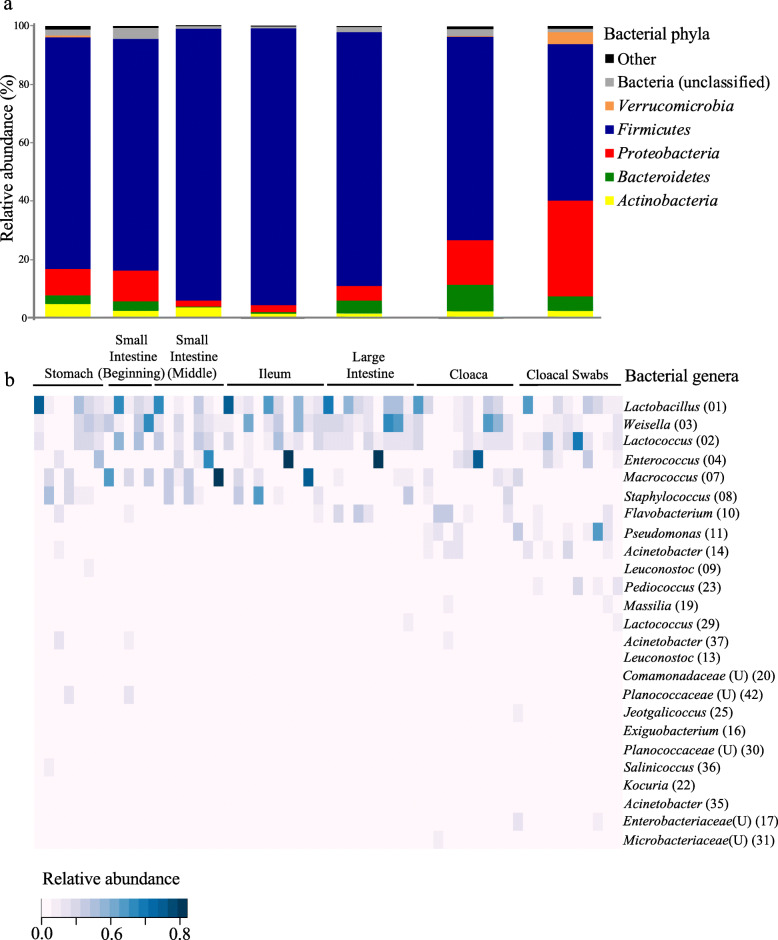
**a** Relative abundances of bacterial phyla in different gut sections and cloacal swabs. **b** Heat map of the relative abundance of the 25 most abundant bacterial OTUs (accounting for a total of 89.5 ± 1.3% (mean ± SE) of sequences in the data set) in different digestive tract regions and cloacal swabs. Genus names provided on the right; U = unclassified genus within the given taxon; OTU numbers given in brackets

Using amplicon sequencing of the 16S rRNA gene, we acquired 1,055,295 (mean ± SE: 17,300 ± 2798) sequences from freshly dissected gut regions and cloacal swabs, which classified into 2189 operational taxonomic units (OTUs) at the 97% similarity level (Additional file 1: Table S1). Bacterial OTU richness (One-way ANOVA: F_6,54_ = 2.556, *p* = 0.029: Fig. 1a) and Chao1 richness estimates (One-way ANOVA: F_6,54_ = 2.445, *p* = 0.0365; Fig. 1b) only differed significantly between cloacal swabs and the cloacal region (Fig. 1a, b). Shannon’s diversity index (One-way ANOVA: F_6,54_ = 1.059, *p* = 0.398; Fig. 1c) and Simpson’s inverse diversity index (One-way ANOVA: F_6,54_ = 1.376, *p* = 0.241; Fig. 1d) did not differ between gut sections and cloacal swabs. Overall, 84.1% of the total number of OTUs belonged to the phylum *Firmicutes*, while *Proteobacteria* accounted for 7.5%, *Bacteroidetes* 3.8% and *Actinobacteria* 2.3%. There was a relative decrease of *Proteobacteria* in the midgut sections (middle of small intestine: 2.2%, ileum: 2.5%, large intestine: 4.8%) compared to the stomach (9.1%), the cloaca (15.2%) and cloacal swabs (32.6%). Compared to the different digestive tract regions, cloacal swabs represented the diversity of the bacterial communities in the whole digestive tract at the phylum level (Fig. 2a). The 25 most common OTUs accounted for 89.5% ± 1.3% (mean ± SE) of the sequences, and cloacal swabs qualitatively represented these major bacterial OTUs from the different digestive tract regions (Fig. 2b).

**Fig. 3:**
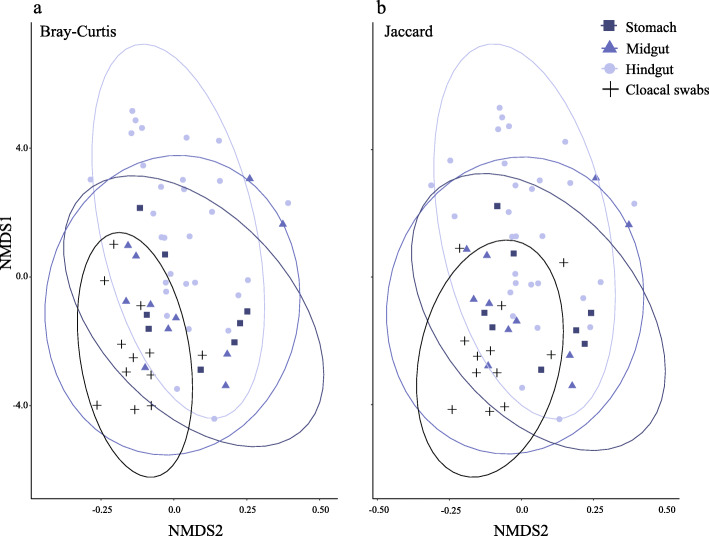
NMDS plots representing gut microbial community similarities based on **a** Bray-Curtis and **b** Jaccard distances of the stomach, the midgut (beginning of small intestine, middle of small intestine, ileum), the hindgut (the large intestine and the cloaca) and cloacal swabs. Gut sections were merged into major gut regions to increase clarity of the ordination. Ellipses represent 95% confidence intervals

We found a significant difference in bacterial community composition between gut sections (including cloacal swabs) [PERMANOVA_999permutations_ (Bray-Curtis): F_6,54_ = 1.82, *R*^2^ = 0.1682, *p* = 0.001]. Community composition did not differ significantly between digestive tract regions; however, cloacal swab bacterial communities differed significantly from four out of six gut regions (Table 1). These community-level differences remained present even after merging results from different gut compartments into three major gut regions (the stomach, the midgut [the small intestine and the cecum], and the hindgut [the large intestine and the cloaca]; Additional file 2: Table S2 and Fig. 3). To qualitatively compare the bacterial communities, we conducted PERMANOVA analyses using the Jaccard distance matrix (based on presence/absence data). Even though the analyses revealed significant qualitative differences in microbial communities among gut sections (including cloacal swabs) [PERMANOVA_999permutations_ (Jaccard): F_6,54_ = 1.44, *R*^2^ = 0.1383, *p* = 0.001], the pairwiseAdonis showed that swabs differed significantly from the large intestine and the ilium (Additional file 2: Table S3).

**Table 1:** The results of pairwiseAdonis analyses (Bray-Curtis distances) between gut microbial communities of different regions of the digestive tract, including cloacal swabs

Comparisons	*F*	*R*^2^	*P* adjusted
Swabs vs. Cloaca	3.576	0.1517	0.0420*
Swabs vs. Large Intestine	3.809	0.1599	0.0210*
Swabs vs. Ilium	4.164	0.1723	0.0210*
Swabs vs. Middle of Small Intestine	2.182	0.1137	0.0630
Swabs vs. Beginning of Small intestine	1.779	0.1128	0.3990
Swabs vs. Stomach	2.425	0.1187	0.0210*
Cloaca vs. Large Intestine	0.6861	0.0367	1
Cloaca vs. Ilium	1.042	0.0547	1
Cloaca vs. Middle of Small Intestine	1.569	0.0946	1
Cloaca vs. Beginning of Small intestine	2.024	0.1443	0.5880
Cloaca vs. Stomach	1.783	0.1003	0.9240
Large Intestine vs. Ilium	0.5702	0.0307	1
Large Intestine vs. Middle of Small Intestine	1.421	0.0865	1
Large Intestine vs. Beginning of Small intestine	1.720	0.1254	1
Large Intestine vs. Stomach	1.440	0.0826	1
Ilium vs. Middle of Small Intestine	0.9653	0.0605	1
Ilium vs. Beginning of Small intestine	1.720	0.1254	0.5880
Ilium vs. Stomach	1.391	0.0799	0.9240
Middle of Small Intestine vs. Beginning of Small intestine	0.8934	0.0903	1
Middle of Small Intestine vs. Stomach	0.6036	0.0444	1
Beginning of Small intestine vs. Stomach	0.8747	0.0804	1

*indicate significantly different groups

### Microbial community composition differences between fresh and alcohol specimens

**Fig. 4:**
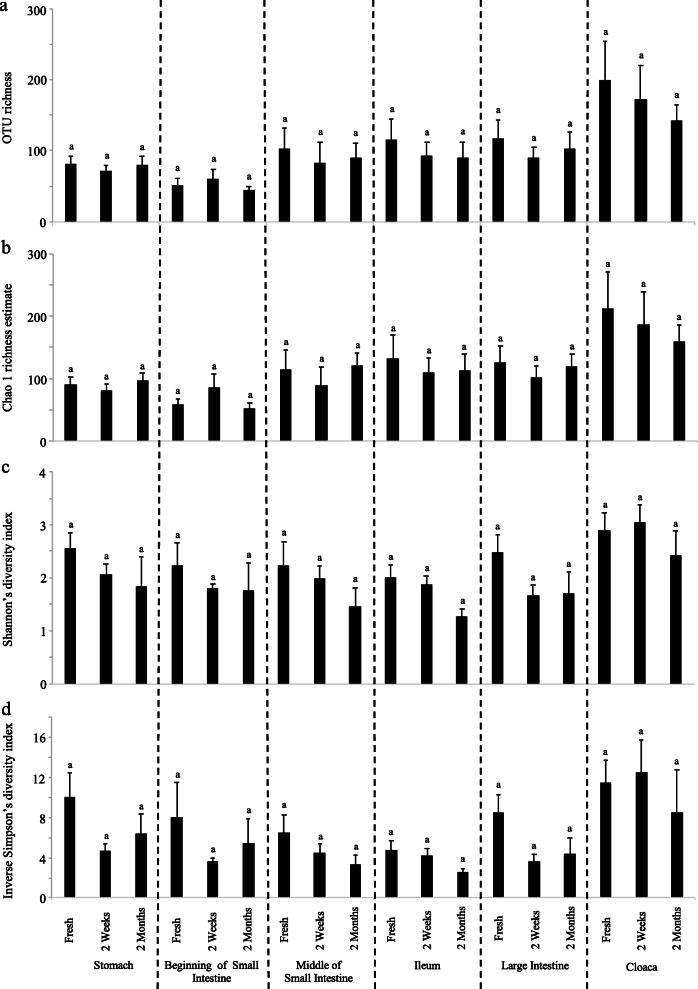
Average (**a**) OTU richness, (**b**) Chao 1 richness estimates, (**c**) Shannon’s diversity index and (**d**) Inverse Simpson’s diversity index of different digestive tract regions of fresh, two-weeks old and two-months old alcohol specimens. Letters above bars indicate the results of Tukey post hoc tests with identical letters representing groups that were not significantly different

**Fig. 5:**
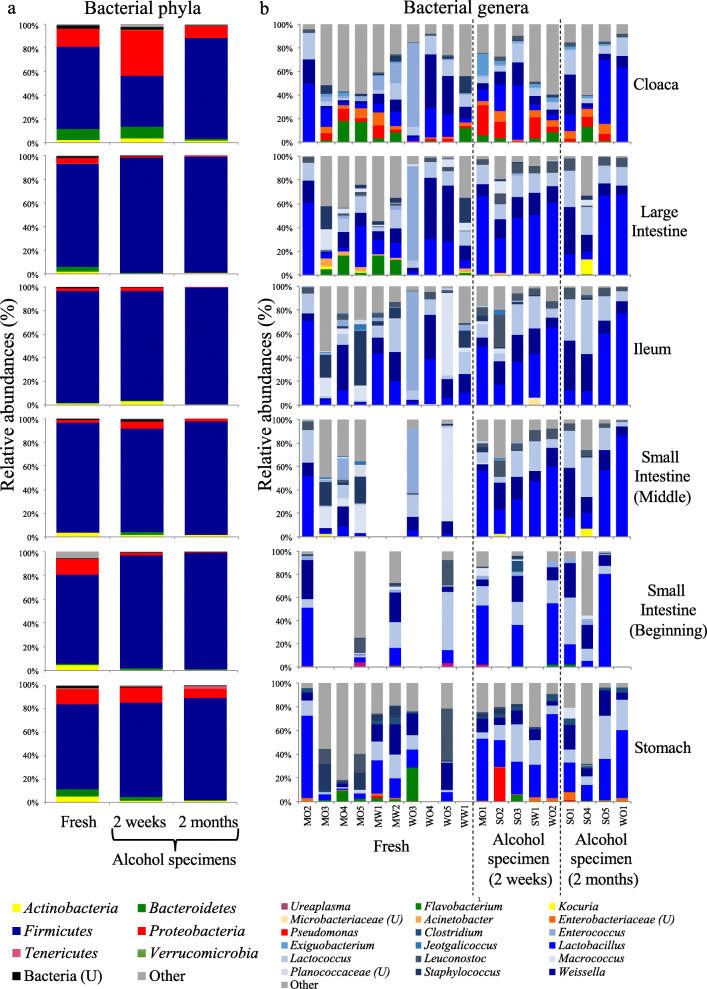
**a** Relative abundance of bacterial phyla in different gut sections preserved under different methods. **b** Relative abundance of the ten most common bacterial genera (accounting for a total of 88.5% of sequences in the data set) across compartments and preserved under different conditions. Each column represents different gut sections from an individual. U = unclassified genera within the given taxon

We generated 1,904,813 16S rRNA amplicon sequences (mean ± SE: 29,048 ± 3057) from the gut sections of bird alcohol specimens, which classified into 2933 OTUs (Additional file 1: Table S1). Similar to the fresh samples, microbial communities of alcohol specimens were dominated by *Firmicutes* (88.0%) followed by *Proteobacteria* (6.16%). OTU richness, Chao1 richness estimate, Shannon’s and inverse Simpson’s diversity indices did not differ in any of the six gut regions between freshly directed samples, and both two-weeks and two-months old alcohol specimens (Fig. 4 and Additional file 2: Table S4). Overall, phylum-level relative abundances of *Bacteroidetes* and *Actinobacteria* decreased in multiple gut sections between fresh and two months old alcohol specimens (Fig. 5a). Despite the high individual variation in gut microbiomes, the 15 most common bacterial genera per gut section were present in both fresh and alcohol specimens (Fig. 5b). The bacterial community compositions of different gut regions did not differ significantly between fresh and alcohol specimens, except between the fresh and two-month old (alcohol specimen) ileal microbiota (Table 2 and Additional file 3: Figure S1).

**Table 2:** Results of Adonis (bold) and pairwiseAdonis (Bray-Curtis distances) analyses of gut microbial communities of digestive tract regions of fresh (0D), two-weeks old (2 W), and two-months (2 M) old alcohol specimen

	Comparison	***F***	***R***^**2**^	***P*** adjust
**Cloaca (df**_**2,18**_**)**		**1.144**	**0.1251**	**0.268**
Pair-wise Adonis	0D vs. 2 W	1.035	0.0794	1
	2 W vs. 2 M	1.173	0.1435	0.720
	0D vs. 2 M	1.479	0.1185	0.477
**Large Intestine (df**_**2,18**_**)**		**1.144**	**0.1251**	**0.245**
Pair-wise Adonis	0D vs. 2 W	0.9088	0.0653	1
	2 W vs. 2 M	1.614	0.1873	0.270
	0D vs. 2 M	0.0866	0.0866	0.993
**Ilium (df**_**2,18**_**)**		**1.579**	**0.1649**	**0.088**
Pair-wise Adonis	0D vs. 2 W	1.326	0.0925	0.549
	2 W vs. 2 M	0.7268	0.0941	1
	0D vs. 2 M	2.247	0.1577	0.108
**Middle of Small Intestine (df**_**2,14**_**)**		**2.562**	**0.2992**	0.003*
Pair-wise Adonis	0D vs. 2 W	1.441	0.1379	0.420
	2 W vs. 2 M	3.815	0.3527	0.06
	0D vs. 2 M	3.097	0.2791	0.021*
**Beginning of Small Intestine (df**_**2,9**_**)**		**1.253**	**0.2637**	**0.219**
Pair-wise Adonis	0D vs. 2 W	1.782	0.2627	0.492
	2 W vs. 2 M	0.8224	0.1705	1
	0D vs. 2 M	1.197	0.1931	0.867
**Stomach (df**_**2,17**_**)**		**1.239**	**0.1418**	**0.198**
Pair-wise Adonis	0D vs. 2 W	1.035	0.0794	1
	2 W vs. 2 M	1.173	0.1435	0.720
	0D vs. 2 M	1.479	0.1185	0.477

*indicates significant differences

**Fig. 6:**
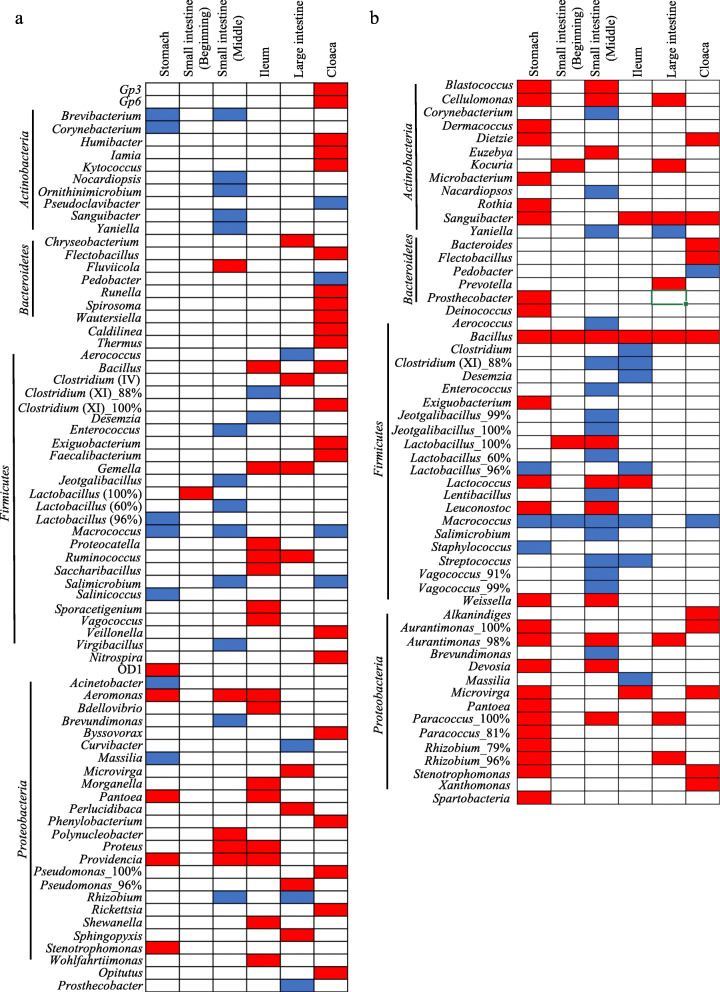
Differentially abundant bacterial taxa at the genus level between different gut sections of (**a**) fresh and two-weeks old and (**b**) fresh and two-months old alcohol specimens. Blue indicates that the taxon was significantly more abundant in fresh samples, and red that the taxon was significantly more abundant in alcohol samples. The figure only represents taxa identified to the genus level (Additional file 4: Table S5)

Multiple DeSeq2 comparisons of microbial communities of freshly dissected and comparable gut sections in differently-aged alcohol specimens revealed only relatively few significantly differentially abundant bacterial genera (Fig. 6 and Additional file 4: Table S5). Only 72 out of 431 genera that were identified to genus-level were significantly differentially abundant in all gut regions between fresh and two-weeks old alcohol specimens, with each gut compartment accounting for on average 14.33 (SE ± 3.09) differentially abundant genera. Between fresh and two-months old alcohol gut sections, total of 55 taxa that identified to genus level were differentially abundant (mean differentially abundant genera per gut section ± SE: 15.16 ± 3.71). Of all differentially abundant genera, only *Bacillus* significantly increased in relative abundance in all gut sections of two-months old alcohol specimens compared to fresh samples, and the genus *Macrococcus* was more abundant in fresh compared to two-months old samples in all gut sections except the large intestine (Fig. 6).

## Discussion

We explored the appropriateness of using cloacal swabs and museum alcohol preserved specimens to investigate the gut microbial communities of wild birds. We first demonstrated that digestive tract regions of wild *P. major* differ in gut microbial communities, but that these differences are not as prominent as previous studies have documented in other bird species [2, 5, 30, 31]. While compositions differed between cloacal swabs and different gut regions, swabs qualitatively portrayed bacterial community compositions in the entire digestive tract, aligning with a previous study of a captive bird species [32], as well as reflected gut community compositions from a previous study of wild *P. major* fecal samples [16]. Microbial communities of alcohol-preserved gut regions did not differ significantly from fresh samples, supporting that museum collections represent a promising resource for analyses of gut microbiomes [37]. Notably, the relative low number of differentially abundant genera in freshly dissected and alcohol preserved specimens only marginally impacted the overall microbial community composition across digestive tract regions.

The possibility of determining the qualitative composition of bird gut microbial communities through cloacal swab sampling support that utilization of this non-invasive method to characterize bird microbiomes is appropriate, despite its quantitative limitations [32, 33]. Cloacal swabs did not capture a few rare bacterial taxa. This is evident from a significant difference only in OTU richness (Fig. 1a and b) but not in diversity indexes that account for both abundance and evenness (Fig. 1c and d). The ability of cloacal swabs to capture the majority of common bacterial taxa (Fig. 2) implies that the method would allow for the vast majority of gut symbionts to be identified. Previous work has suggested that fecal sampling better represents gut community compositions than cloacal swabs [2]. Although we did not compare fecal samples with cloacal swabs, the bacterial taxa we acquired from cloacal swabs were similar to taxa characterized in fecal microbiomes of wild *P. major* in a previous study [16], suggesting that microbiome discrepancies between these sampling methods are minor*.* However, the cloacal swab approach also reduces the risk of contamination and removes the problems associated with the typically lower DNA yield from extractions of bird feces [42, 43].

The observed similarity of gut microbial communities in fresh and alcohol preserved specimens opens of for the possibility of utilizing the vast number of museum bird alcohol specimens [37]. Although we did observe high individual variation in microbial communities, which is common in bird gut microbiomes [13, 16, 37, 44], the most common bacterial genera did not differ significantly between preservation methods (Fig. 4). The slight reduction in the relative abundance of members of the phyla *Bacteroidetes* and *Actinobacteria* in alcohol preserved specimens could be due to degradation of bacterial DNA. In addition, it may be that ethanol adversely affects DNA extraction, but a previous study suggests that this has a minimal impact on microbial community characterizations [4]. The genera that were differentially abundant between alcohol preserved and fresh specimens were inconsistent across different gut regions, suggesting that they are unlikely to be caused by the alcohol preservation, but rather are due to the naturally high variation between individuals in passerine bird gut microbiomes [16, 37]. Overall, alcohol preserved specimens thus provide a reliable estimate of gut microbial communities, even after two months of storage.

The utilization of museum specimens to investigate wild bird gut microbiomes would allow substantial insights into bird-microbial symbiosis in multiple ways. First, museum collections provide an opportunity to investigate gut microbiomes of a vast number of extant and extinct wild bird species [38–41] to improve our understanding of the ecology and evolution of birds and their gut microbial symbionts. Secondly, museum collections usually also include individuals from multiple geographic regions, allowing insights into host-symbiont biogeography. Finally, collections typically include a long temporal span, enabling investigations into how bird-gut microbial associations change over time. This would allow us to establish natural temporal changes in associations, but also to test for the effects of climate change and habitat alterations caused by anthropogenic effects. Notably, most museum alcohol specimens are older than two months and longer-term effects of preservations on gut microbiome will thus also be needed. New specimen collections should ideally also focus on preserving the entire animal in alcohol (instead of focusing solely on skins and skeletons) and in storage conditions optimal for the possibility of using these collections for microbiome studies [38].

## Conclusions

Our findings demonstrate that cloacal swabs and museum alcohol specimens are reliable to qualitatively characterize gut microbiome compositions in wild birds. Both methods have limitations but also the potential to markedly improve our understanding of symbioses between wild birds and their gut microbial communities. The ease of cloacal sampling reduces issues related to low sample sizes or sampling from threatened species and may thus help strengthen both population and community-level host microbiome studies. The similarity of gut microbiomes of alcohol preserved and fresh samples provides the possibility of using underexplored museum bird collections to investigate gut microbiomes of a plethora of bird species across the globe and time.

## Methods

### Sample collection

Nineteen adult individuals of *P. major* (captured as fledglings in Ceske Budejovice, the Czech Republic) were kept in individual cages on a daily standard diet consisting of 10 mealworms, 2 g insect cake [45] a bread-like diet made from - Nutribird a21, commercial chicken food (Country’s Best Show 1 crumble), eggs, wheat flower, sugar and sunflower margarine, mixed and baked for ~ 40 min at ~ 180 °C temperature] and 2tsp of moistened mixed seeds (Living World Premium Mix for Cockatiels & Lovebirds) at the Faculty of Science, University of South Bohemia, Ceske Budejovice, Czech Republic. Cages were cleaned and individuals were given fresh water and food daily. One day before the euthenization of individuals to extract the entire digestive tract or converting them into alcohol specimen, cloacal swabs were collected using a mini FLOQ swabs™ (Copan, Italy) and stored in RNAlater® at − 21 °C. Cloacal swabs were acquired from the individuals 1 day prior to the euthenization to ensure that the cloacal swab sampling did not have an impact on the microbiome of freshly dissected and alcohol preserved cloacal regions. Individuals were euthanized using CO_2_ chambers, following the guidelines of the Czech Republic’s dispensation of the law no. 359/2012 Col., § 17, par. 1 (i.e., animal cruelty act). *P. major* were captured and raised in captivity under the permit number OOZP/5345/2018/R La issued by Environmental Protection Department and were euthanized under the permit number MZP/2018/785/1363 issued by the Ministry of the Environment of the Czech Republic. Digestive tracts from 10 randomly chosen individuals were dissected immediately after euthenization on a sterile surface and separated into six main regions (stomach, beginning of the small intestine, middle of the small intestine, ileum including ceca, large intestine, cloaca). Gut sections were stored in RNAlater at − 21 °C until DNA extractions.

The remaining nine birds were preserved as whole specimens in 70% ethanol. Of these alcohol specimens, five were dissected after two weeks and four were dissected after two months. After dissection, the gut sections were stored in RNAlater at − 21 °C until the DNA extractions. After the acquisition of digestive tract all the specimens are deposited in the alcohol bird collections of the Natural History Museum of Denmark, Copenhagen, Denmark (For NHMID numbers, see Additional file 2: Table S6).

### Molecular methods

Prior to DNA extractions, individual gut sections were mixed thoroughly using sterile pestles and 100 μl of this homogeneous mixture was used for the DNA extractions. The entire tip of the swab along with 100 μl of RNAlater was used to extract DNA from the cloacal swabs. DNA was extracted using Qiagen blood and tissue DNeasy kits (Qiagen, Germany), following the manufactures guidelines, except that we added mini glass beads during the lysis step to increase the physical lysis of bacterial cells and we incubated the samples (along with proteinase K and ATL buffer) for 12 h at 56 °C. We also used 75 μl heated (50 °C) elution buffer to elute the DNA.

Initial PCRs were conducted using SA511 and SB701 primers targeting the v4 region of the 16S rRNA gene to identify the samples with bacterial DNA (cf. [37]). PCR reactions contained total of 25 μl (1 μl of reverse primer, 1 μl forward primer, 12.5 μl of VWR red Taq® polymerase, 8.5 μl of MilliQ water and 2 μl of DNA) and PCRs were conducted under an initial denaturing condition of 96 °C for 4 min, followed by 35 cycles of denaturing (94 °C for 30 s), annealing (56 °C 30 s) and extension (72 °C for 30 s) and an a final extension step of 72 °C for 4 min. DNA from the successfully amplified samples (after visual inspection of products on a 2% agarose gel) were sent to the Microbial System Molecular Biology Lab at the University of Michigan for MiSeq amplicon sequencing (using the same primers) on an Illumina platform.

### Data analysis

MiSeq sequences were analyzed using mothur 1.35.1 [45]. Chimeric and non-prokaryotic sequences were removed following the mothur pipeline. Cleaned sequences were aligned and identified using the SILVA 132 database [46]. Samples containing less than 1000 sequences were removed from the analysis. Downstream analyses were conducted in R 3.5.3 [47, 48]. Alpha diversity (OTU richness, Chao 1 richness estimate, Shannon diversity index and inverse Simpson’s diversity index), Permutational multivariate analysis of variance (PERMANOVA) based on Bray-Curtis (with abundances) and Jaccard distance matrixes (presence/absence of OTUs), and non-matric multidimensional scaling (NMDS) analyses were conducted using the vegan package [49]. For pair-wise PERMANOVAs, we used the wrapper package pairwiseAdonis [50]. Tukey HSD post hoc tests were conducted to investigate pairwise statistically significant differences in alpha diversity between digestive tract regions (including cloacal swabs) and gut regions under different storing conditions. DeSeq2 [51] was utilized within the MicrobiomeSeq package [52] along with Phyloseq package [53] to investigate significantly differentially abundant bacterial genera between samples.

## Supplementary information

**Additional file 1: Table S1.** OTU table of all samples, categorized according to the sample region and preservation method (freshly dissected, two-weeks old and two-month alcohol specimens).

**Additional file 2: Table S2.** Results of pairwiseAdonis analysis between gut microbial communities of major gut regions [the stomach, the midgut (the small intestine and the cecum), and the hindgut (large intestine and cloaca)] and cloacal swabs. Asterisks indicate sections that were significantly different in composition. **Table S3.** The results of pairwiseAdonis analyses using Jaccard distances between gut microbial communities of different regions of the digestive tract (including cloacal swabs). **Table S4.** Summary of one-way analysis of variance (ANOVA) on OTU richness (R), Chao1 richness estimate (C), Shannon’s diversity index (Sh) and Inverse Simpson’s diversity index (Isimp) of freshly dissected gut sections and alcohol preserved specimens. **Table S6.** Museum ID numbers (Natural History Museum of Denmark), sexes and treatments (freshly dissected, two-weeks old alcohol specimens and two-months old alcohol specimens) of the 19 *P. major* used in this study.

**Additional file 3: Figure S1.** NMDS plots of microbial community similarities between different gut sections of fresh and differently aged alcohol samples. Ellipses represent 95% confidence intervals.

**Additional file 4: Table S5.** Results from the DeSeq2 analysis on differentially abundant bacterial genera between fresh and differently aged alcohol specimens. Each tab includes data from one region of the digestive tract.

## Data Availability

The datasets generated and analyzed during the current study are available in the GenBank SRA repository (SRA accession: PRJNA591550, Bio sample accession numbers [gut sections]: SAMN13383021 – SAMN13383134, Cloacal swab accession numbers: SRR9295628, SRR9295605, SRR9295561, SRR9295668, SRR9295635, SRR9295590, SRR9295610, SRR9295576, SRR9295676, SRR9295651, SRR9295639, SRR9295585).
