# Supplementary material for: Cloacal swabs and alcohol bird specimens are good proxies for compositional analyses of gut microbial communities of Great tits (Parus major)
Source: Anim Microbiome. 2020 Mar 17;2:9. doi: 10.1186/s42523-020-00026-8 (PMC7807456; doi:10.1186/s42523-020-00026-8)
Supplement: Supplementary file 2 — Additional file 2: Table S2. Results of pairwiseAdonis analysis between gut microbial communities of major gut regions [the stomach, the midgut (the small intestine and the cecum), and the hindgut (large intestine and cloaca)] and cloacal swabs. Asterisks indicate sections that were significantly different in composition. Table S3. The results of pairwiseAdonis analyses using Jaccard distances between gut microbial communities of different regions of the digestive tract (including cloacal swabs). Table S4. Summary of one-way analysis of variance (ANOVA) on OTU richness (R), Chao1 richness estimate (C), Shannon’s diversity index (Sh) and Inverse Simpson’s diversity index (Isimp) of freshly dissected gut sections and alcohol preserved specimens. Table S6. Museum ID numbers (Natural History Museum of Denmark), sexes and treatments (freshly dissected, two-weeks old alcohol specimens and two-months old alcohol specimens) of the 19 P. major used in this study. [file 42523_2020_26_MOESM2_ESM.docx]

**Supplementary figures and tables**

Cloacal swabs and alcohol bird specimens are good proxies for compositional analyses of gut microbial communities of Great tits (*Parus major*)

Kasun H. Bodawatta^1^, Katerina Puzejova^2,3^, Katerina Sam^2,3^, Michael Poulsen^4^ and Knud A. Jønsson^1^

^1^Natural History Museum of Denmark, University of Copenhagen, Copenhagen, Denmark

^2^Biology Centre of Czech Academy of Sciences, Institute of Entomology, Ceske Budejovice, Branisovska 31, Czech Republic

^3^Faculty of Science, University of South Bohemia, Ceske Budejovice, Branisovska 1760, Czech Republic

^4^Section for Ecology and Evolution, Department of Biology, University of Copenhagen, Copenhagen, Denmark

Corresponding author: Kasun H. Bodawatta: email – [bodawatta@snm.ku.dk](mailto:bodawatta@snm.ku.dk), T. P. - +45 91 72 49 96

**Supplementary tables 2, 3, 4 and 6**

Table S2. Results of pairwiseAdonis analysis (Bray-Curtis distances) between gut microbial communities of major gut regions [the stomach, the midgut (the small intestine and the cecum), and the hindgut (large intestine and cloaca)] and cloacal swabs. Asterisks indicate sections that were significantly different in composition.

|  | F | R^2^ | P adjusted |
| --- | --- | --- | --- |
| Swabs vs. End | 5.398 | 0.1189 | 0.006* |
| Swabs vs. Midgut | 2.492 | 0.1061 | 0.006* |
| Swabs vs. Stomach | 2.425 | 0.1187 | 0.012* |
| Hindgut vs. Middle | 2.530 | 0.0609 | 0.061 |
| Hindgut vs. Stomach | 1.846 | 0.0488 | 0.336 |
| Midgut vs. Stomach | 0.6302 | 0.0357 | 1 |

Table S3. The results of pairwiseAdonis analyses on Jaccard distances between gut microbial communities of different regions of the digestive tract (including cloacal swabs).

| Comparisons | F | R^2^ | P adjusted |
| --- | --- | --- | --- |
| Swabs vs. Cloaca | 2.437 | 0.1086 | 0.0630 |
| Swabs vs. Large Intestines | 2.608 | 0.1154 | 0.0210* |
| Swabs vs. Ilium | 2.716 | 0.1195 | 0.0210* |
| Swabs vs. Middle of Small Intestine | 1.616 | 0.0868 | 0.2310 |
| Swabs vs. Beginning of Small intestine | 1.497 | 0.0966 | 0.2940 |
| Swabs vs. Stomach | 1.809 | 0.1187 | 0.0840 |
| Cloaca vs. Large Intestine | 0.7312 | 0.0914 | 1 |
| Cloaca vs. Ilium | 0.9293 | 0.0491 | 1 |
| Cloaca vs. Middle of Small Intestine | 1.328 | 0.0814 | 1 |
| Cloaca vs. Beginning of Small intestine | 1.593 | 0.1172 | 0.9240 |
| Cloaca vs. Stomach | 1.444 | 0.0827 | 1 |
| Large Intestine vs. Ilium | 0.6365 | 0.0342 | 1 |
| Large Intestine vs. Middle of Small Intestine | 1.284 | 0.0789 | 1 |
| Large Intestine vs. Beginning of Small intestine | 1.462 | 0.1086 | 1 |
| Large Intestine vs. Stomach | 1.262 | 0.0731 | 1 |
| Ilium vs. Middle of Small Intestine | 0.9121 | 0.0605 | 1 |
| Ilium vs. Beginning of Small intestine | 1.547 | 0.1142 | 0.6300 |
| Ilium vs. Stomach | 1.104 | 0.645 | 1 |
| Middle of Small Intestine vs. Beginning of Small intestine | 0.9285 | 0.0935 | 1 |
| Middle of Small Intestine vs. Stomach | 0. 6987 | 0.0511 | 1 |
| Beginning of Small intestine vs. Stomach | 0.9259 | 0.0847 | 1 |

Table S4. Summary of one-way analysis of variance (ANOVA) on OTU richness (R), Chao1 richness estimate (C), Shannon’s diversity index (Sh) and Inverse Simpson’s diversity index (Isimp) of freshly dissected gut sections and alcohol preserved specimens.

|  | df | F | p value |
| --- | --- | --- | --- |
| Stomach (R) | 2,15 | 0.173 | 0.843 |
| Stomach (C) | 2,15 | 0.251 | 0.781 |
| Stomach (Sh) | 2,15 | 0.982 | 0.398 |
| Stomach (Isimp) | 2, 14 | 1.339 | 0.294 |
| Beginning of small intestine (R) | 2,7 | 0.392 | 0.689 |
| Beginning of small intestine (C) | 2,7 | 1.032 | 0.405 |
| Beginning of small intestine (Sh) | 2,7 | 0.319 | 0.737 |
| Beginning of small intestine (Isimp) | 2,7 | 10.496 | 0.629 |
| Middle of small intestine (R) | 2,12 | 0.101 | 0.905 |
| Middle of small intestine (C) | 2,12 | 0.277 | 0.763 |
| Middle of small intestine (Sh) | 2,12 | 0.901 | 0.432 |
| Middle of small intestine (Isimp) | 2,12 | 1.024 | 0.389 |
| Ileum (R) | 2,16 | 0.192 | 0.827 |
| Ileum (C) | 2,16 | 0.109 | 0.897 |
| Ileum (Sh) | 2,16 | 1.768 | 0.202 |
| Ileum (Isimp) | 2,16 | 1.14 | 0.344 |
| Large intestine (R) | 2,16 | 0.253 | 0.779 |
| Large intestine (C) | 2,16 | 0.168 | 0.847 |
| Large intestine (Sh) | 2,16 | 1.593 | 0.234 |
| Large intestine (Isimp) | 2,16 | 2.064 | 0.159 |
| Cloaca (R) | 2,16 | 0.221 | 0.805 |
| Cloaca (C) | 2,16 | 0.162 | 0.852 |
| Cloaca (Sh) | 2,16 | 0.451 | 0.645 |
| Cloaca (Isimp) | 2,16 | 0.303 | 0.743 |

Table S6. Museum ID numbers (Natural History Museum of Denmark), sexes and treatments (freshly dissected, two-weeks old alcohol specimens and two-months old alcohol specimens) of the 19 *P. major* used in this study.

| Individual code name (from the experiment) | NHMID number | Sex | Treatment |
| --- | --- | --- | --- |
| MO2 | 307498 | Female | Freshly dissected |
| MO3 | 307497 | Male | Freshly dissected |
| MO4 | 307496 | Female | Freshly dissected |
| MO5 | 307495 | Female | Freshly dissected |
| MW1 | 307494 | Female | Freshly dissected |
| MW2 | 307493 | Female | Freshly dissected |
| WO3 | 307492 | Female | Freshly dissected |
| WO4 | 307491 | Male | Freshly dissected |
| WO5 | 307490 | Female | Freshly dissected |
| WW1 | 307489 | Male | Freshly dissected |
| MO1 | 307488 | Female | Two-weeks old alcohol specimen |
| SO2 | 307487 | Female | Two-weeks old alcohol specimen |
| SO3 | 307486 | Female | Two-weeks old alcohol specimen |
| SW1 | 307485 | Female | Two-weeks old alcohol specimen |
| WO2 | 307484 | Female | Two-weeks old alcohol specimen |
| SO1 | 307483 | Male | Two-months old alcohol specimen |
| SO4 | 307482 | Female | Two-months old alcohol specimen |
| SO5 | 307481 | Female | Two-months old alcohol specimen |
| WO1 | 307480 | Male | Two-months old alcohol specimen |
