# Supplementary figures and images for: Cloacal swabs and alcohol bird specimens are good proxies for compositional analyses of gut microbial communities of Great tits (Parus major)
Source: Anim Microbiome. 2020 Mar 17;2:9. doi: 10.1186/s42523-020-00026-8 (PMC7807456; doi:10.1186/s42523-020-00026-8)

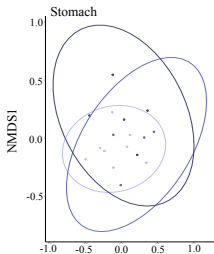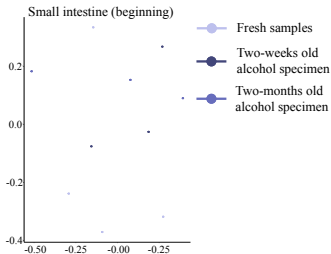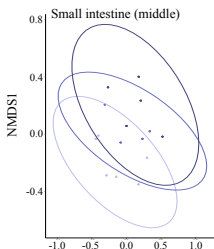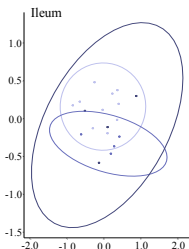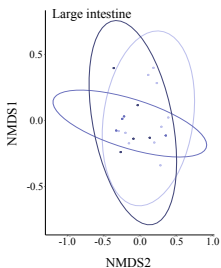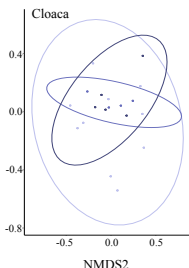

Supplement: Supplementary file 3 — Additional file 3: Figure S1. NMDS plots of microbial community similarities between different gut sections of fresh and differently aged alcohol samples. Ellipses represent 95% confidence intervals. [file 42523_2020_26_MOESM3_ESM.pdf]
